# Macrophages in MASLD: from inflammatory and metabolic crosstalk to exercise intervention

**DOI:** 10.3389/fimmu.2026.1894884

**Published:** 2026-07-09

**Authors:** Yi Sun

**Affiliations:** 1 Key Laboratory of Adolescent Health Assessment and Exercise Intervention, Ministry of Education, East China Normal University, Shanghai, China; 2 College of Physical Education and Health, East China Normal University, Shanghai, China

**Keywords:** exercise, hepatic macrophage, inflammation, macrophage polarization, MASLD, metabolic crosstalk

## Abstract

Metabolic dysfunction-associated steatotic liver disease (MASLD) places a heavy burden on public health, with no approved therapies. Hepatic macrophages are central to MASLD pathogenesis. In this review, the clinical spectrum of MASLD and the heterogeneity of liver macrophages are summarized, covering classical M1/M2 polarization and novel subsets identified by single-cell transcriptomics. Beyond the well-recognized inflammatory roles, non-inflammatory mechanisms through which macrophages contribute to insulin resistance and steatosis are also highlighted. Importantly, a novel framework is established, which positions exercise as a systemic integrator. In this framework, exercise simultaneously dampens adipose-derived pro-inflammatory inputs while amplifying muscle-derived protective signals, thereby rewiring multi-organ communication to reprogram hepatic macrophage function. Finally, evidence is integrated, showing that exercise re-polarizes macrophages, reshapes intrahepatic immune landscape, and mediates muscle-liver communication through exercise-induced factors. Together, this review provides a framework for understanding macrophage-centered mechanisms in MASLD, and highlights lifestyle intervention as a promising therapeutic strategy.

## 1 Introduction

The prevalence of metabolic diseases has increased at an exponential rate over the past few decades (1). Hepatic steatosis (fatty liver) is one of the most prevalent chronic liver diseases worldwide. Accumulating evidence has suggested that obesity is closely linked to metabolic dysfunction-associated steatotic liver disease (MASLD). The public health burden because of MASLD as well as its comorbidities has become a big issue. However, even though strong efforts have been made to bring up new ways to treat or alleviate MASLD, there are still no agreed therapies (2). MASLD is a heterogeneous disorder. It only draws attention when clinical signs such as liver damage, inflammation and fibrosis are manifested, which also means that steatosis has progressed to metabolic dysfunction-associated steatohepatitis (MASH) (3). Hepatocellular carcinoma (HCC) and cirrhosis are two further progressed diseases that develop from MASH due to inflammatory mechanisms (3). However, simple steatosis does not always progress to MASH, and the mechanisms remain largely unknown. In this context, hepatic macrophages have become key orchestrators of MASLD pathogenesis, bridging metabolic stress, inflammation, and fibrosis (4, 5).

Hepatic macrophages include embryonically derived Kupffer cells (KCs) and monocyte-derived macrophages (MoMFs), which are recruited from the circulation (6, 7). In healthy conditions, KCs preserve liver homeostasis by performing immune surveillance and eliminating gut-derived antigens (6). During MASLD progression, however, the hepatic macrophage pool undergoes profound remodeling. The resident KCs (ResKCs) exhibit dysfunctional self-renewal, while infiltrating MoMFs accumulate and adopt distinct phenotypic states (8, 9). Single-cell transcriptomics has further revealed the heterogenous nature of hepatic macrophages, revealing specialized subsets named lipid-associated macrophages (LAMs) and scar-associated macrophages (SAMs) (10, 11).

Beyond the liver, macrophages in other metabolic organs also contribute to MASLD pathogenesis through inter-organ crosstalk (12). In obesity, adipose tissue macrophages (ATMs) switch towards more pro-inflammatory phenotype, and release cytokines and extracellular vesicles that reach the liver and exacerbate steatohepatitis (13). Skeletal muscle has also emerged to be an active endocrine organ, which communicates with the liver via myokines and muscle-derived extracellular vesicles (EVs), modulating hepatic macrophage function and metabolic outcomes (14). Lifestyle interventions, particularly exercise, have been shown to ameliorate MASLD partly through modulating macrophage polarization (15). Exercise induces the release of anti-inflammatory myokines and promotes M1/M2 phenotypic switching in both liver and ATMs, offering a non-pharmacological strategy to combat metaflammation (16).

Here, we first summarize the clinical manifestations and pathophysiology of MASLD, and then focus on the inflammatory aspects of MASLD, especially the role and heterogeneity of macrophages. We also discuss the non-inflammatory participation of macrophages in MASLD, as well as the interplay between the liver, adipose tissue and skeletal muscle. Finally, we conclude by discussing the potential impact of exercise intervention in the treatment of MASLD.

## 2 The spectrum of MASLD

The liver is an important metabolic organ, possessing remarkable biological functions and regenerative capabilities. In insulin resistance, the low capacity of adipose tissue to store fat triggers excessive lipid accumulation, resulting in fatty liver (17). The lipid overload overwhelms the liver, leading to aberrant lipid peroxidation and excessive production of reactive oxygen species (ROS)/reactive nitrogen species (RNS) (18). In 1842, William Bowman for the first time proposed fatty degeneration of the liver through observing human liver species under microscope (19). Forty years ago, Dr. Fenton Schaffner proposed the term nonalcoholic fatty liver disease (NAFLD) to describe the prevalence of a fatty liver without significant alcohol consumption (1, 20). Over time, the term “NAFLD” has changed. The essential metabolic aspect of hepatic steatosis may be better described by the name “metabolic dysfunction-associated steatotic liver disease (MASLD),” according to a consensus panel of specialists in 2023 (21). Throughout this review, we uniformly adopt the MASLD/MASH terminology, even when citing older literature that originally used NAFLD/NASH, in order to maintain consistency and to reflect the current understanding of the disease’s metabolic underpinnings.

**Figure 1:**
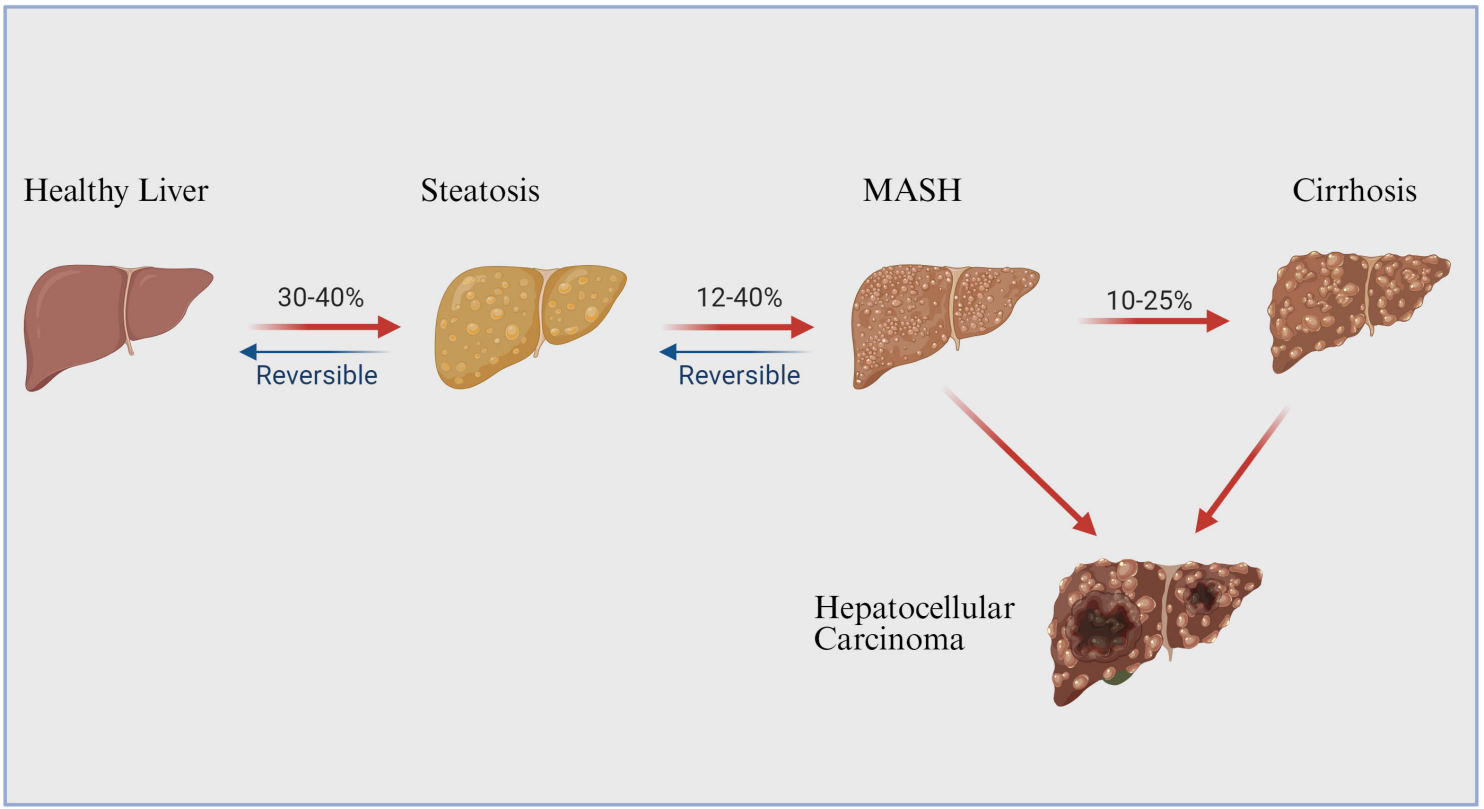
Spectrum of MASLD. The progression from healthy liver to simple steatosis, and from steatosis to MASH, is reversible (bidirectional arrows). In contrast, the transition from MASH to cirrhosis and from cirrhosis to HCC is generally irreversible. The percentages indicate approximate proportions of patients advancing to the next stage. MASLD affects 30-40% adults globally. About 12-40 % of individuals with steatosis progress to MASH. Among those with MASH, 10–25 % develop cirrhosis. Histological scoring remains gold standard for diagnosis and risk stratification across the MASLD spectrum. Created in BioRender. Sun, Y. (2026) https://BioRender.com/9x18m61.

MASLD is a spectrum of progressive liver diseases. Steatosis, which means excessive lipid buildup within hepatocytes, marks the initial stage of disease. Hepatic steatosis, hepatocellular destruction, lobular inflammation, and fibrosis are features of the more severe variant, metabolic dysfunction-associated steatohepatitis (MASH) (1). Although steatosis is observed in MASLD, it does not always lead to MASH. Lipotoxicity, oxidative stress, and inflammation are among the additional pressures to cause MASH onset. These stresses work together to trigger cellular stress pathways that cause hepatocyte death, inflammation, and the development of fibrosis. Hepatic fibrosis, a common pathology in liver diseases, results from extracellular matrix (ECM) buildup triggered by HSC activation and their conversion to myofibroblast-like cells. Patients with severe fibrosis and MASH may develop cirrhosis and hepatocellular carcinoma (HCC). MASH is therefore a crucial stage in the clinical progression of MASLD. The spectrum of MASLD is illustrated in **Figure 1**.

## 3 Introduction of macrophages

Macrophages are a type of immune cells, and are present in all vertebrate tissues. They are widely recognized for engulfing big particles. Tissue macrophages form a distributed cellular system: the mononuclear phagocyte system (MPS) (22). The term “macrophage” derives from Greek words *makrós phagein*, meaning “large eaters” (22, 23). During development, tissue-resident macrophages are produced by erythromyeloid progenitors that are distributed across tissues from the embryonic yolk sac and fetal liver (22). Tissue-resident macrophages are fundamental to the function of the innate immune system. These macrophages undergo local turnover and carry out trophic and clearing tasks. Blood monocytes, which replenish resident macrophage populations with high turnover, originate from bone marrow hemopoietic stem cells around the time of birth. After an injury, infection, or inflammation, these monocytes are recruited and develop into infiltrating active tissue macrophages (22).

Numerous factors can be produced by macrophages, including cytokines, chemokines and growth factors. Macrophages were initially appreciated for fighting infection, chronic inflammatory diseases and cancer. In addition, increasing evidence describes macrophages as being essential to reaching and preserving metabolic homeostasis. They can adapt to environmental changes, nutrients, and long-term systemic alterations (24). Thus, the role of macrophages in metabolic disorders like type 2 diabetes, insulin resistance, lipid dyslipidemia, obesity and MASLD has been thoroughly investigated over the last 20 years. Macrophages frequently constitute a major fraction of the leukocyte population linked to metabolic diseases (25), making them a significant component in the developing fields of immunometabolism (26) and metaflammation (27).

## 4 Types of hepatic macrophages

The initial line of defense against substances from the gastrointestinal system is provided by the large number of hepatic macrophages (28). Tissue ResKCs and MoMFs make up liver macrophages (7). Usually found on the luminal side of the liver sinusoids, liver macrophages partially protrude into the perisinusoidal region, where they contact closely with HSCs (29). With single-cell RNA sequencing (scRNA-seq) technique, it is now possible to define macrophage populations (23).

### 4.1 Macrophage polarization (M1/M2 paradigm)

Macrophages can switch between various phenotypes, and show distinct heterogeneity (4). Local cytokines direct the functional specialization of macrophages, resulting in a phenomenon known as “macrophage polarization”. According to a widely accepted model, macrophages are classified as either pro-inflammatory M1 or anti-inflammatory M2 (30). Type I cytokines (e.g., IFN-γ, TNF-α) along with damage-associated molecular patterns (DAMPs) and pathogen-associated molecular patterns (PAMPs) trigger classical M1 activation, leading to exacerbated tissue damage. Alternatively activated M2 macrophages mainly help to dampen inflammation and support tissue repair. They reduce inflammation by phagocytosing apoptotic cells and secreting mediators, such as TGF-β, VEGF, and EGF that promote tissue remodeling and regeneration. M2a, M2b and M2c are additional classifications for alternatively activated M2 macrophages (31). However, the traditional classification as M1/M2 has been challenged by single-cell transcriptomic studies, and the latter one could reveal the full range of macrophage functions in tissues crucial to metabolic homeostasis. In both embryo-derived macrophages and MoMFs, the most regulated genes are those related to lipid metabolism (8).

### 4.2 Kupffer cells and their heterogeneity

KCs are an essential part of hepatic macrophages. In a heathy liver, embryo-derived KCs make up around 95% of resident macrophages. These cells are thought to be long-lived, and rely mainly on local self-renewal for maintenance (1). Functionally, KCs serve as the liver’s first line of immune defense, performing immune surveillance, scavenging gut-derived antigens, and maintaining hepatic immune tolerance (32, 33). Except KCs, a small proportion of MoMFs also reside in the healthy liver. A study using scRNA-seq based on healthy human livers discovered two populations of intrahepatic CD68^+^ macrophages (34). MARCO (Macrophage Receptor with Collagenous structure) is only expressed in non-inflammatory KCs. Therefore, human CD68^+^ MARCO^+^ cells are transcriptionally similar to ResKCs in mouse, have a tolerogenic function and express elevated levels of VCAM1, CD5L, HMOX1, MRC1, CD163, M4SA7, and VSIG4. CD68^+^ MARCO^-^ macrophages express less CD163, and exhibit transcriptional signatures resembling those of inflammatory, recruited macrophages. Analysis of transcriptomes of human liver cells also showed that KCs express CD163 and MARCO (10).

### 4.3 Monocyte subsets and recruitment

**Figure 2:**
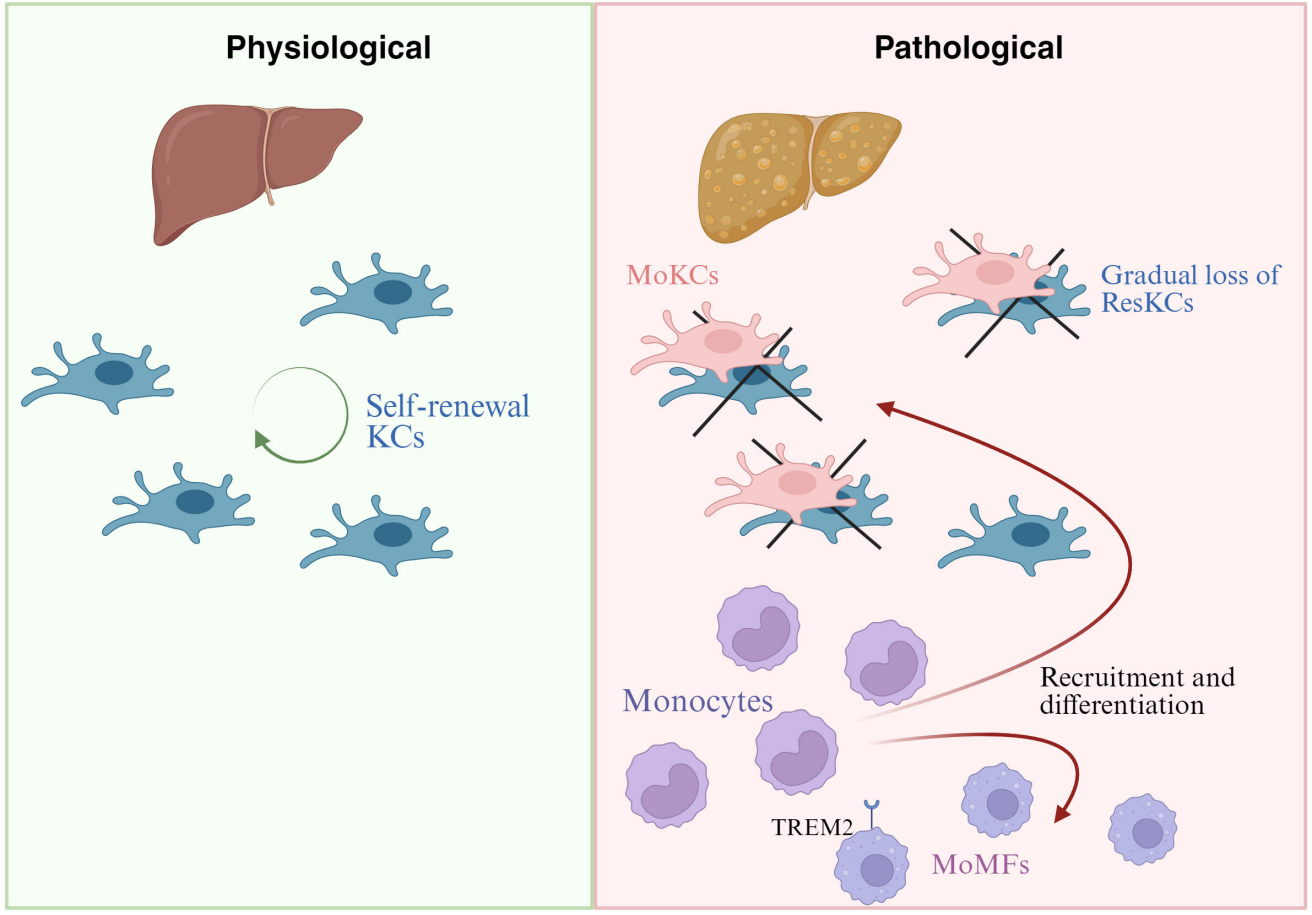
Hepatic macrophage remodeling in physiological versus pathological conditions. In the healthy liver, the macrophage pool is dominated by resident Kupffer cells (ResKCs), which maintain long-term self-renewal as well as perform immune surveillance, clearance of gut-erived antigens, and homeostatic functions. In pathological conditions, the hepatic macrophage landscape undergoes profound remodeling. ResKCs exhibit dysfunctional self-renewal and increased death. In response, circulating monocytes differentiate into macrophages once recruited into the liver. Monocyte-derived KCs (MoKCs) partially replenish the KC niche but display a more pro-inflammatory phenotype. Monocytes can also differentiate into monocyte-derived macrophages (MoMFs). These TREM2^+^ macrophages accumulate in steatotic and fibrotic regions, and are involved in lipid handling, clearance of apoptotic cells, and fibrosis regulation. The shift from a self-sustaining ResKCs pool to a monocyte-derived and TREM2^+^-enriched macrophage compartment contributes to persistent inflammation, metabolic dysregulation, and disease progression in MASLD. Created in BioRender. Sun, Y. (2026) https://BioRender.com/9x18m61.

According to scRNA-seq studies, KCs constitute the majority of liver macrophages in mice (23). KCs are known to prolong inflammation by releasing pro-inflammatory cytokines/chemokines and activating HSC. However, hepatic inflammation is only partially conducted by KCs, and is largely dependent on recruitment of monocytes. Monocytes serve as circulating precursors for macrophages. The liver is very accessible to monocytes because of the high volume of blood that passes through. During KCs depletion, inflammation or metabolic diseases, bone marrow-derived monocytes (BMDMs) differentiate into self-renewing KCs in the presence of an available niche, and gradually adopt the transcriptional profile of KCs (35). There is heterogeneity among human monocytes (36). Three subsets of monocytes exist in peripheral blood: CD14^++^CD16^-^monocytes, CD14^dim^CD16^++^ monocytes and CD14^+^CD16^+^ monocytes (36, 37). CD14^++^CD16^-^ monocytes, also termed classical monocytes, comprise 95% of monocytes in the healthy individuals. CD14^+^CD16^+^ monocytes are called intermediate monocytes, and CD14^dim^CD16^++^ monocytes are also known as non-classical monocytes. Different subsets of monocytes also exist in mice (38). Murine inflammatory Ly6C^hi^ monocytes equate to human CD14^++^CD16^-^ monocytes, while murine patrolling Ly6C^lo^ monocytes share functional similarities to human CD14^dim^CD16^++^ monocytes (39, 40). **Figure 2** illustrates hepatic macrophage remodeling in physiological versus pathological conditions.

### 4.4 Scar-associated macrophages (SAMs) and other novel subsets

A subpopulation, which is named scar-associated macrophages (SAMs) was originally found in human liver (10). These macrophages express unique markers including triggering receptor expressed on myeloid cells 2 (TREM2) and CD9, and display a hybrid phenotype of both tissue monocytes and KCs. TREM2^+^ CD9^+^ macrophages were confirmed to expand in human fibrotic livers. These human SAMs are analogous to MoMFs in mouse liver (41). A study based on mouse reversible hepatic fibrosis model identified CD11B^hi^ F4/80^int^ Ly-6C^lo^ macrophage subset as most abundant in fibrotic liver, and show a phenotype other than M1/M2 classification (41). Including SAMs, studies have identified three unique but similar populations of TREM2-expressing macrophages in MASLD, termed by different names based on their contexts and disease stages. The other two are lipid associated macrophages (LAMs) (42) in steatotic livers and NASH-associated macrophages (NAMs) (10) in fibrotic livers. It is worth noting that LAMs, SAMs and NAMs were initially defined in three independent studies published in 2019, each describing TREM2^+^ macrophages in different disease contexts. LAMs were first identified in the adipose tissue of obese mice and humans as a subset that responds to lipid homeostasis (42). SAMs were discovered in human cirrhotic livers and exhibit a pro-fibrogenic phenotype (10). NAMs were uncovered in mouse and human MASH livers, and were linked to disease severity (43). Therefore, these three designations likely refer to overlapping populations, which share a convergent pattern of gene expressions (44). The distinct names reflect tissue context, disease stage and focus of the original studies, rather than indicating mutually exclusive cell subsets. Actually, researchers tend to use LAMs, SAMs and NAMs interchangeably in literatures to describe TREM2^+^ macrophages in MASLD (45).

It is important to clarify that traditional M1/M2 classification and the novel single-cell-derived subsets (LAMs, SAMs and NAMs) represent two complementary yet distinct classification systems. The M1/M2 paradigm describes functional polarization states based on cytokine profiles and effector functions (30). In contrast, the novel subsets are defined by transcriptomic profiling, ontogeny and spatial localization within specific hepatic niches (10, 46). More importantly, these novel subsets do not strictly conform to the M1/M2 dichotomy. For example, LAMs express certain M2-associated markers (such as CD206), but often lack the canonical M2 enzyme Arg1 (47). Therefore, LAMs and SAMs should be viewed as distinct differentiation states that transcend the classical binary framework (44, 48). Given the complexity of hepatic macrophage heterogeneity across different classification systems, the major subpopulations identified by various approaches are summarized in **Table 1**.

**Table 1:** **Overview of** hepatic macrophage subsets in MASLD.

Classification	Subpopulation	Markers	Functions and characteristics	References
Classical M1/M2 paradigm	M1 (classically activated)	iNOS, CD86, CD16, CD32, MHCII	Pro-inflammatory, Th1 immune response, antigen presentation, anti-tumor	Chen 2023 (30), Van Ginderachter 2026 (31), Moehle 2015 (171)
	M2a (alternatively activated)	Arg-1, CD206, CD163, SRs, Fizz1	Anti-inflammatory, tissue repair, Th2 immune regulation	
	M2b	CD86, MHCII	Immunoregulatory; mixed pro- and anti-inflammatory properties	
	M2c	CD163, CD206, SLAM	Anti-inflammatory, immunosuppression, phagocytosis of apoptotic cells, matrix remodeling	
Ontogeny and spatial niche	Embryo-derived KCs	CLEC4F^+^, CLEC2, TIMD4, F4/80^high^, CD163, MARCO	Self-renewing, liver resident, homeostatic maintenance, immune surveillance, die in MASH, efficiently promote triglyceride storage	Tran 2020 (8), Ramachandran, 2019 (10)
	Monocyte-derived KCs	Ly6C^+^ to CLEC4F^+^, CLEC2	Monocyte-derived in MASH, gradually replenish the KC pool, pro-inflammatory phenotype, exacerbate liver injury	Tran 2020 (8), Tacke 2017 (6)
	Monocyte-derived macrophages	CCR2^+^, Ly6C^high^, CD14^+^	Infiltrating, amplify inflammation, pro-fibrotic, do not acquire KC phenotype	Krenkel 2018 (172), Tacke 2017 (6)
Novel subsets defined by single-cell transcriptomics	LAMs (lipid associated macrophages)	TREM2^+^, CD9^+^, Gpnmb^+^, CD36^+^, FABP5^+^, SPP1^+^	Respond to loss of lipid homeostasis, lipid handling, clearance of apoptotic cells, located in steatotic regions, dual pro-inflammatory and repair functions	Jaitin 2019 (42), Daemen 2021 (48)
	SAMs (scar-associated macrophages)	TREM2^+^, CD9^+^, SPP1^+^	Located in fibrotic scars, monocyte-derived, pro-fibrogenic, expand in cirrhosis	Ramachandran, 2019 (10)
	NAMs (NASH-associated macrophages)	Trem2^+^, Cd9^+^, Gpnmb^+^,	Monocyte-derived in MASH, partial loss of KC identity, pro-inflammatory; pro-fibrotic	Xiong 2019 (43)
	Transitional infiltrating macrophages	Cx3cr1^+^, CCR2^+^	Transitional subset infiltrating the liver, expand during MASH progression	Daemen 2021 (48)

## 5 Liver macrophages and MASLD

Macrophage contribution to MASLD pathogenesis has been characterized extensively. During MASH, the hepatic migration of blood monocytes is driven by inflammatory cues. These monocytes locally develop into MoMFs, enlarging the macrophage pool of the liver.

### 5.1 Inflammatory role of liver macrophages in MASLD

Simple steatosis is considered as the first hit to initiate MASLD. However, a single hit is insufficient to cause fibrosis, and a second hit is needed to worsen liver damage as the disease advances (49). According to the “multiple hits” hypothesis, hepatic inflammation rather than steatosis, is the main driver of progression from MASH to fibrosis (50, 51). Chronic inflammation is a dynamic process wherein hepatic macrophages exhibit a dual role (4). Macrophage activation and polarization are initiated by their interaction with DAMPs in the early phase of liver injury. The activated macrophages then generate cytokines with pro-inflammatory and profibrogenic properties, thereby promoting fibrosis. In addition, macrophages also play a role in fibrosis resolution, indicated by the observation that depletion of macrophages hinders fibrosis regression.

**Figure 3:**
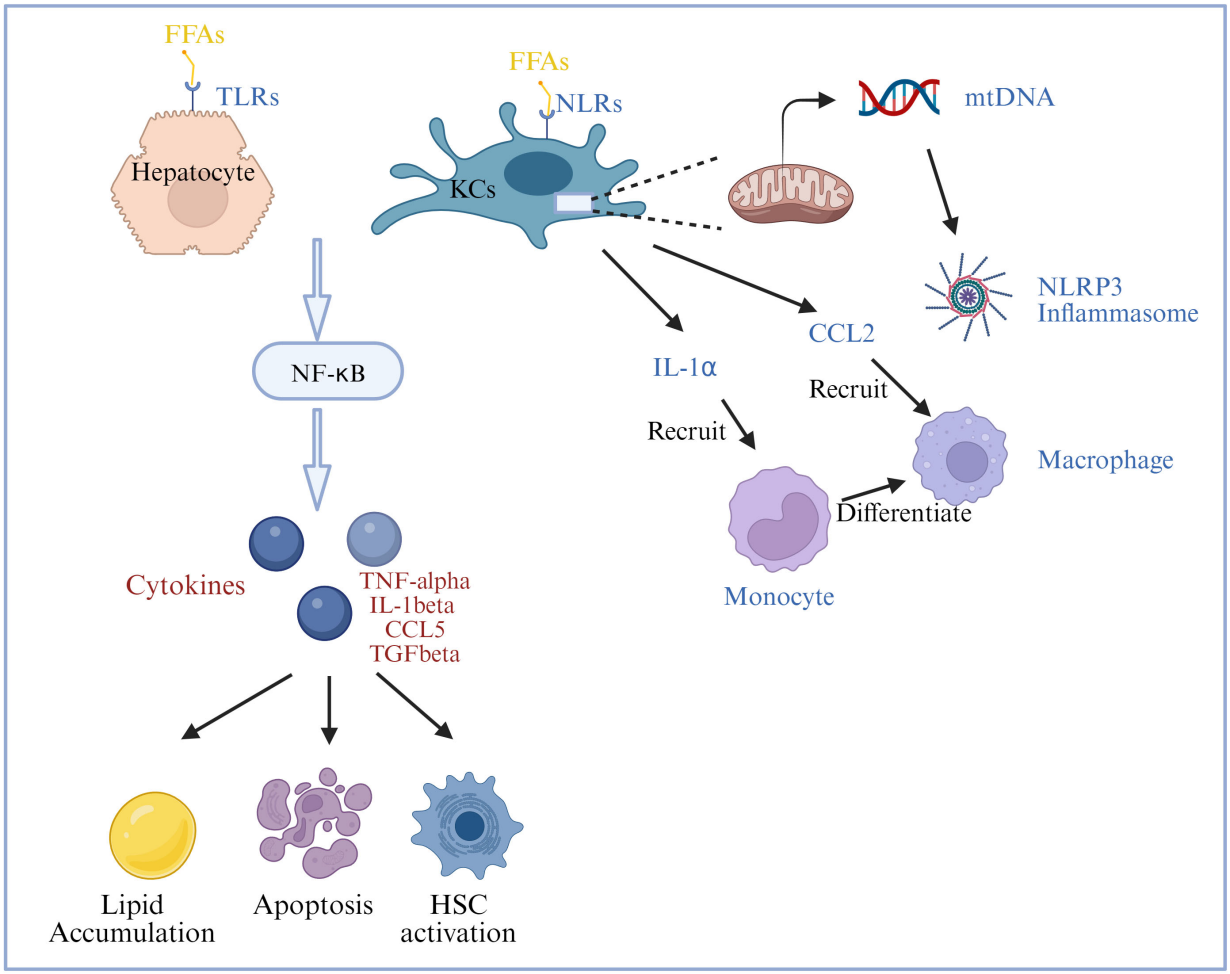
Inflammatory signaling in Kupffer cells drives MASLD progression. In response to danger signals (HMGB1, FFAs, mtDNA, cholesterol crystals), Hepatocytes and Kupffer cells activate TLRs and NLRs, leading to NF-κB-mediated production of pro-inflammatory and pro-fibrotic cytokines (TNF-α, IL-1β, CCL5, TGF-β) and NLRP3 inflammasome-dependent IL-1β maturation. These mediators promote hepatocyte injury, hepatic stellate cell activation, and recruitment of monocyte-derived macrophages via CCL2/CCR2, amplifying inflammation and fibrosis. Created in BioRender. Sun, Y. (2026) https://BioRender.com/9x18m61.

KCs are essential to the development of MASLD, acting as the liver’s primary sensing system (52). Hepatic Toll-like receptors (TLRs) and NOD-like receptors (NLRs) on KCs are activated by endogenous substrates including HMGB1 and free fatty acids (FFAs). Downstream, nuclear factor kappa-B (NF-κB) becomes activated, leading to elevated release of pro-inflammatory and profibrogenic cytokines, including TNF-α, IL-1β, CCL5, and TGFβ. Outcomes include hepatocyte lipid accumulation, hepatocyte apoptosis, and HSC activation (9). Once activated, HSCs transdifferentiate into myofibroblast-like cells and become the principal collagen-producing cells responsible for extracellular matrix (ECM) deposition and fibrosis progression (53). Additionally, KC-driven IL-1α upregulation recruits monocytes to inflammatory foci, amplifying hepatic injury. Thus, IL-1 has emerged as a promising intervention point for MASH. IL-1Ra attenuates inflammation by blocking IL-1α/IL-1β from activating the IL-1R signaling pathway (54). In KCs, fatty acids stimulate the release of mtDNA and cholesterol crystals, which activate NOD-like receptor family pyrin domain containing 3 (NLRP3) inflammasome (55). Additionally, by secreting CCL2, activated KCs facilitate the CCR2-dependent recruitment of MoMFs to the liver (28). The recruited inflammatory MoMFs drive further inflammatory escalation. **Figure 3** illustrates the inflammatory signaling pathway in KCs under MASLD.

EVs have emerged as critical signaling mediators that propagate inflammatory signals between hepatocytes and macrophages in MASLD. Inositol-requiring enzyme 1 alpha (IRE1A) acts as a sensor of unfolded protein response, and is activated in MASH livers. In mice with diet-induced MASH, activated IRE1A results in ceramide biosynthesis and release of EVs, which then recruits MoMFs to the liver, causing hepatic inflammation and injury (56). Another critical player is hepatocyte-derived exosomal miR-192-5p, which strongly promotes M1 macrophage activation and liver inflammation (57). Consistent with this, MASLD patients exhibit serum miR-192-5p levels that correlate positively with the degree of hepatic inflammatory activity. To be more specific, miR-192-5p-enriched exosomes originating from hepatocytes activate pro-inflammatory macrophages via Rictor-Akt-FoxO1 signaling (57).

Oxidative stress and insulin resistance have been linked to increased hepatic macrophage proliferation (58). During the initial phases of insulin resistance, the overall quantity of liver macrophages remain unchanged (8). However, dysfunctional self-renewal of resident liver macrophages was still observed (59). During MASH, there is an increase in the death of embryonically derived KCs (8). Monocyte-derived KCs are then generated aiming at maintaining KC numbers. Indeed, fate-mapping studies show that monocytes feed into the mouse KC pool in MASH (8). Immunostaining experiments confirm that these monocyte-derived KCs settle in the sinusoids of the liver, like their embryonic counterparts (59). However, monocyte-derived KCs have a distinct inflammatory profile and an altered metabolic phenotype compared to their embryonically derived counterparts (8). While the KC pool in the liver is maintained by monocyte-derived KCs, these cells still show a distinct transcriptional profile from embryonic KCs (8, 59, 60). For instance, unlike their embryotic counterparts, monocyte-derived KCs do not fully express genes linked to roles such as erythrophagocytosis, and they tend to be more inflammatory (8). During MASH, a fraction of KCs is derived from Ly6c^high^ monocytes. These monocyte-derived KCs are partly immature and gradually seed the KC pool with disease progression. Notably, while KCs as a whole favor hepatic triglyceride storage during MASH, embryo-derived KCs promote this function more efficiently than monocyte-derived KCs, and the latter exacerbate liver damage and fibrosis (58). Thus, MASH leads to substantial disruption of KC homeostasis, which affects liver pathology. A recent study revealed that monocyte-to-macrophage transition was regulated by Notch-RBPJ signaling during MASH, providing a potential therapeutic target (61). Another study demonstrated that NCF1 governs ferroptosis susceptibility of KCs in MASH by regulating reactive oxygen species production, highlighting a novel mechanism of KC dysfunction (62).

Apart from replenishing the KC pool, monocytes in MASH also undergo differentiation, and produce monocyte-derived inflammatory macrophages. ScRNA-seq revealed that distinctive gene signature and inflammatory phenotype were both observed in liver macrophages as well as their precursors in bone marrow (63). High expressions of Spp1, Itgax, Gpnmb, Cd9 and Trem2 are observed in MoMFs in the liver (59, 60). A certain level of these markers has also been seen in monocyte-derived KCs (8). Different subsets of hepatic macrophages in MASLD have also been examined, and four subsets were identified, including ResKCs, monocyte-derived KCs, pre-monocyte-derived KCs and hepatic LAMs (59). All four subsets occupied the KC niche, where they contact HSCs and liver sinusoidal endothelial cells (LSECs). In particular, LAMs were preferentially located in areas of higher Desmin expression, indicative of liver fibrosis. This finding aligns with observations in human liver cirrhosis, where TREM2^+^CD9^+^ MoMFs accumulates (10). However, in a mouse study, LAMs showed markedly reduced mRNA levels of IL-1β and TNF-α following 24 weeks of Western diet. Together, these findings suggest that LAMs participate in both early pro-inflammatory damage and late anti-inflammatory repair phases in MASH (64). Tsomidis et al. provides an comprehensive review of the involvement of KCs and MoMFs across all stages of MASLD pathogenesis (65).

### 5.2 Cell death and MASLD

Beyond the inflammatory roles discussed above, hepatocyte cell death and its interaction with macrophages constitute another key mechanism in MASLD progression. Hepatocytes are the predominant parenchymal cells in the liver, accounting for approximately 70-80% of liver volume, and are central to hepatic lipid metabolism, gluconeogenesis, and cholesterol homeostasis (66). In MASLD, prolonged overnutrition leads to excessive accumulation of triglycerides and toxic lipid intermediates within hepatocytes, disrupting metabolic functions and rendering them susceptible to cell death (17). Apoptosis of hepatocytes commonly drives liver disease progression in mice as well as in human (64). The severity of MASH correlates with a marked increase in hepatocyte apoptosis. Immunohistochemistry showed active caspase 3 and caspase 7 in MASH, confirming apoptosis involvement in the disease (67, 68). In addition, both pro-apoptotic Bax and anti-apoptotic Bcl-2 are strongly expressed, reflecting adaptive response to MASH-related stress (68).

Liver steatosis derives from either adipose lipolysis-released FFAs or hepatic *de novo* lipogenesis (69). Saturated FFAs can induce JNK-dependent heptocyte apoptosis through the engagement of proapoptotic Bcl-2 proteins (70). Additionally, evidence showed mitochondrial membrane depolarization together with cytochrome c release, suggesting trigger of mitochondrial apoptotic pathway. Lipid-mediated stress enhances the susceptibility of hepatocytes to apoptosis, thereby contributing to liver injury. In mice, high-fat diet (HFD) feeding has shown to sensitize hepatocytes to cytokine-induced cell death (71). These dying hepatocytes then activate macrophages through releasing DAMPs and lipid messengers (1). Therefore, hepatocyte lipotoxicity and inflammation form a feed-forward relationship (72). Lipotoxicity triggers the release of EVs from hepatocytes. Once these vesicles are engulfed by macrophages, macrophages are activated and start to express Fas ligand (FasL), TNF-related apoptosis-inducing ligand (TRAIL) and TNF-α. This further exacerbates inflammation and hepatocyte apoptosis. To the contrary, TREM2^+^ macrophages protect against MASLD by limiting pro-inflammatory responses and engulfing apoptotic cells (71).

TREM2^+^ macrophages play a protective role during MASLD progression. TREM2^+^ macrophages prevent pro-inflammatory response and are involved in clearing apoptotic cells via phagocytosis. Although apoptosis itself triggers a relatively mild inflammatory response during MASLD progression, the extensive release of TNF-α and TGF-β from activated macrophages can provoke additional cell death, collectively promoting MASLD pathogenesis (49).

### 5.3 Non-inflammatory role of liver macrophages in MASLD

Even though it is well accepted that macrophages are mainly involved in MASLD via inflammatory pathways, accumulating evidence show that macrophages also contribute to insulin resistance and hepatic steatosis via inflammation-independent mechanisms. These non-inflammatory pathways can be broadly categorized into four distinct mechanisms discussed below.

#### 5.3.1 NF−κB downstream effectors

It is well established that liver macrophages play a role in metabolic diseases through producing inflammatory cytokines. Nevertheless, anti-inflammatory agents have yielded marginal benefits in systemic metabolism, pointing to the existence unidentified non-inflammatory drivers (73). A study investigating this possibility placed mice on HFD for 9 weeks, after which the mice developed obesity, steatosis, and insulin resistance, yet monocyte recruitment did not occur until three weeks later. This temporal dissociation, where insulin resistance and steatosis precede inflammation in obese mouse livers, suggests that liver macrophages may disrupt metabolism without requiring inflammatory activation (74). Further evidence comes from loss-of-function experiments. Silencing NF-κB in KCs lowered IL-1β expression and improved insulin sensitivity in obese mice (75). However, silencing IL-1β in liver macrophages failed to replicate this effect, implying that NF-κB’s beneficial actions could be inflammation-independent (73). Indeed, liver macrophage-derived insulin-like growth factor-binding protein 7 (IGFBP7) provides direct evidence for such non-IL-1β factors. IGFBP7 binds to the insulin receptor and activates the ERK pathway, driving lipogenesis and gluconeogenesis (73). Therefore, macrophages can promote insulin resistance regardless of their inflammatory status.

#### 5.3.2 Oxidative stress and antioxidant activity

Another inflammation-independent pathway involves oxidative stress within macrophages. A shift in liver macrophage phenotype, from M2 to M1, is shown to be triggered by ROS and RNS, causing insulin resistance (76). Studies have also described liver macrophages as the major origin of ROS. However, despite lacking a pro-inflammatory phenotype during obesity, liver macrophages could still exhibit oxidative stress (77). The same study further showed that nuclear factor erythroid 2-related factor 2 (NRF2), an antioxidant factor, was decreased in mouse and human livers, compromising response to lipid accumulation. In addition, silencing of miR-144 could reverse this effect by restoring NRF2 levels and antioxidant activity. The above findings highlight the role of macrophages in endogenous antioxidant response during MASLD progression.

#### 5.3.3 Metabolic intermediates

In addition to oxidative stress, metabolic intermediates are also known to regulate macrophage function in MASLD. MASH represents a disruption of metabolic crosstalk among hepatocytes, macrophages and HSCs. An excess of fatty acids, together with impaired mitochondrial oxidation, causes intermediates to accumulate. These metabolites activate inflammation, creating a vicious cycle that further impairs metabolism. MASLD involves a significant enhancement of glycolytic activity, the hallmark of which is increased lactate. Elevated hexokinase 2 (HK2) and histone H3 lysine 18 lactylation (H3K18la) are detected in hepatic macrophages from MASLD patients and mice (78). Histone lactylation then promotes glycolysis and hepatic M1 polarization by promoting transcription of glycolytic genes. Eventually, HK2, glycolysis and H3K18la forms a positive feedback loop, exacerbating metabolic dysfunction and inflammation in liver macrophages. In addition, targeting macrophage lactate uptake via inhibiting monocarboxylate transporter 1 (MCT1) could reduce inflammation and fibrosis in mouse MASH model (79). Similar effect has been observed by targeting lactate production via blocking lactate dehydrogenase or hexokinase 2 (78, 80). Lactylation has also been linked to HSCs activation in MASH (81), confirming that lactate acts as a link between hepatocyte immunometabolic activation and HSC-mediated fibrosis (82).

Succinate, as an intermediate of TCA cycle, seems another emerging regulator of inflammation and fibrosis. Both liver macrophages and HSCs express succinate receptor 1 (SUCNR1, GPR91) (83). In mice, choline-deficient (MCD) diet caused enhanced steatohepatitis and liver fibrosis. Treatment with FGF21 analogue decreased succinate level, GPR91 expression and α-SMA production in the liver, suggesting that inhibiting succinate-GPR91 pathway relieves liver fibrosis (84). Succinate also promote inflammation and macrophage recruitment through stabilization of hypoxia-inducible factor 1-alpha (HIF-1α), causing production of IL-1β, TNF-α and IL-6, thereby acting as a bona fide metabolite regulator of innate immune signaling (85, 86).

Besides lactate and succinate, other metabolic intermediates also emerged as direct regulators of macrophage polarization. Creatine has been shown to reprogram macrophage polarization by suppressing M1 while promoting M2 effector functions through modulation of STAT1 phosphorylation and chromatin accessibility (87). Sarcosine, a glycine derivative, activates the GCN2 signaling pathway to enhance anti-inflammatory macrophage polarization, thereby promoting adipose thermogenesis and muscle regeneration (88). These findings reveal that metabolic intermediates not only serve as energy substrates, but also active signaling molecules that shape macrophage functional states.

#### 5.3.4 Nuclear receptor signaling

In addition to the direct signaling roles of succinate and lactate, the transcriptional machinery that governs macrophage metabolism and inflammation is also subject to modulation by nuclear receptors, notably peroxisome proliferator-activated receptors (PPARs). These receptors are members of the nuclear hormone receptor superfamily, and function as transcriptional factors activated by ligands. Three PPAR isotypes have been identified (α, β/δ, and γ), each contributing to lipid and glucose metabolism in the context of MASLD (49). PPARα serves as the main regulator of hepatic fat catabolism during fasting (89). Experimental evidence shows that PPARα deficiency promotes MASLD and hepatic inflammation in mice (90), and HFD-fed PPARα^-^/^-^ mice exhibit marked hepatic lipid accumulation (91). Liver transcriptomic analysis reveals that PPARβ/δ deletion suppresses pathways involved in lipoprotein and glucose metabolism, while enhancing expression of genes related to inflammation. These changes collectively cause elevated plasma glucose and TG (92). Some liver macrophages adopt an anti-inflammatory phenotype via PPARδ (93), and these cells can also promote apoptosis of inflammatory liver macrophages through caspase 3 (94). Of note, recent evidence indicates that exogenous succinate exposure suppresses AMP-activated protein kinase (AMPK)/PPARα/FGF21-dependent fatty acid oxidation, thereby promoting hepatic triglyceride accumulation and steatosis in nonobese MASLD models (95).

Given the multiple inflammation-independent pathways described above (IGFBP7, oxidative stress, lactate/succinate signaling, and PPARδ-mediated phenotype switching), it is not surprising that current clinical therapies targeting classical inflammatory cascades have shown only partial efficacy. Even though substantial progress has been observed with multiple therapies in phase III clinical trials for MASH, only a subset of individuals respond. Therefore, maybe agents such as thyroid hormone receptor β (THRβ), glucagon-like peptide-1 (GLP-1) receptor, fibroblast growth factor 21 (FGF-21) receptor and PPAR agonists have therapeutic potentials against inflammation and fibrosis of the liver (82). However, it requires further investigation regarding their effects on the crosstalk between hepatocytes, macrophages and HSCs, and how these therapies influence the handling of specific fatty acid species. Therefore, future studies should focus on how lactate and succinate dynamically regulate PPAR isoform activity across hepatocytes, macrophages, and HSCs, and whether targeting the metabolite-nuclear receptor axis can overcome the limitations of current anti-inflammatory therapies in MASH.

### 5.4 Liver macrophage populations in MASLD: focus on lipid-associated macrophages (LAMs)

While the previous section briefly introduced LAMs as part of the dynamic inflammatory landscape during MASH progression, this section provides a focused analysis of the origin, differentiation, and functional characterization of this distinct macrophage subset.

LAMs are specialized macrophage subset often found in metabolic diseases, adapting to lipid-rich environments. They are almost absent in healthy livers (46, 96), but can be found in diseased lesions. LAMs mainly differentiate from circulating Ly6C^+^ monocytes rather than from ResKCs. However, the process is strongly impaired in CCR2-knockout mice, where LAMs fail to appear and hepatic crown-like structures (hCLSs) do not form (48, 97). In MASLD, LAMs account for a substantial portion of the MoMFs (48). They are generated when the steatotic regions in murine and human livers are exposed to local lipids, as demonstrated by Guilliams et al. using a spatial proteogenomic atlas (11). Therefore, the liver contains two main monocyte-derived pathways: MoKC pathway and LAMs pathway. Notably, LAMs may also acquire certain KC surface markers, and remain present for a long time following disease regression (8).

LAMs precursors found in the livers of obese mice and humans express monocyte-specific genes, such as CCR2 and Cx3cr1, which promote monocyte migration and aggregation (48). Single-cell transcriptome studies have revealed a pre-LAMs intermediate differentiation stage marked by Ms4a7 expression. This stage is characterized by intermediate expressions of monocytic genes, and an initial upregulation of LAMs-associated genes before acquiring mature phenotype (48). LAMs share some similarities with both M1 and M2 macrophages. However, their pro-inflammatory activity is far weaker than classical M1 macrophages. Therefore, LAMs are considered an independent functional subset outside the M1/M2 dichotomy (97, 98). In addition, scar-associated macrophages (SAMs), which resemble LAMs, localize to fibrotic scars in human cirrhosis and mouse steatohepatitis (10). SAMs share core markers with LAMs such as TREM2^+^, CD9^+^ and SPP1^+^, and are further enriched in fibrosis-related genes.

LAMs manifest distinct functions depending on the disease stage of MASLD. During MASH progression, LAMs largely contribute to inflammation and fibrogenesis. A unique CD36^+^ LAM subset expands markedly in MASLD livers (99). Macrophage-specific CD36 deletion attenuated steatosis and fibrosis in MASH models. To be specific, CD36 facilitates lipid transfer from steatotic hepatocytes to LAMs, activating PPARγ-SPP1 axis, which drives HSC activation and fibrogenesis. In MASH regression, LAMs maintain high TREM2 expression. These TREM2^+^ macrophages are functionally critical for restricting both fibrosis and inflammation (100). Investigation on the role of TREM2 in MASH regression shows that absence of TREM2 restricts the emergence of LAMs and formation of hCLSs, causing impaired collagen degradation, lipid clearance and HSCs inactivation (100). TREM2^+^ macrophages are also involved in regulating hepatic lipid metabolism. When TREM2 is absent, exosomes released by macrophages become overabundant with miR-106b-5p (101). Once engulfed by hepatocytes, this miRNA suppresses mitofusin-2 (Mfn2), thereby compromising mitochondrial function and aggravating lipid deposition.

## 6 Liver-adipose tissue crosstalk and MASLD

Adipose tissue is critical to maintaining energy and immune homeostasis. Excessive adiposity is a significant risk factor for type 2 diabetes, MASLD and other cardiometabolic diseases (102). Uncontrolled expansion of adipose tissue causes reduced adipogenesis, adipocyte hypoxia and chronic low-grade inflammation. Therefore, adipose tissue inflammation has evolved as a crucial player in the pathogenesis of MASLD (103). Normal adipose tissue is composed of adipocytes, fibroblasts, endothelial cells, resident macrophages and other cells of the immune system.

### 6.1 Adipose tissue dysfunction in MASLD

In obesity, adipose tissue inflammation is characterized by increased expressions of cytokines and chemokines, as well as infiltration of immune cells. As a result, the inflammatory state of adipose tissue drives system inflammation, aggravating insulin resistance and MASLD (12). Under healthy conditions, adipose tissue communicates with the liver to regulate whole-body homeostasis (104, 105). The recruitment of ATMs relies on chemokines such as CCL2 (106). Nevertheless, direct evidence linking such crosstalk to liver macrophages remains limited (23). In one study, patients with MASH showed increased CD11c^+^CD206^+^ macrophages in visceral adipose tissue (VAT), as well as enhanced production of pro-inflammatory cytokines and chemokines (107). As regards to studies using tissue-specific knockout models, adipose-specific knockout of insulin receptor and IGF1 receptor induces severe lipid dysregulation and MASH-like symptoms, characterized by inflammation and fibrosis (108). In addition, AT transplantation from obese mice to lean mice caused increased liver injury and hepatic inflammation (109). However, hepatic inflammation and injury was less pronounced when AT was transplanted from ATM-depleted obese mice (109).

### 6.2 Heterogeneity of adipose tissue macrophages

Macrophages represent the most abundant cell type in the stromal vascular fraction of adipose tissue, and respond to lipid excess and inflammatory signals, thereby modulating insulin resistance (110). During obesity, progressive accumulation of adipose tissue macrophages (ATMs) contribute to metabolically dysfunctional adipose tissue. To be more specific, ATMs produce pro-inflammatory cytokines, such as IL-6, TNF-α and IL-1β, which further impair adipocyte function and promote system metabolic dysfunction (102). The formation of crown-like structures (CLS), where macrophages surround dying adipocytes, is a characteristic of obese adipose tissue and is strongly associated with MASLD severity (111). Similar to hepatic macrophages, ATMs were also traditionally classified into pro-inflammatory M1-like and anti-inflammatory M2-like phenotypes. However, this binary classification is oversimplified and fails to capture the full complexity of ATM functions in metabolic diseases. The development of single-cell transcriptomis has led to the discovery of multiple distinct ATM subsets (110).

ATM subsets include metabolically active macrophages (MMacs) (112), resident vasculature-associated macrophages (ResVAMs) (112) and lipid-associated macrophages (LAMs) (42), among which LAMs have emerged as a prominent subset in obese adipose tissue. Adipose LAMs show high expressions of markers such as TREM2, Cd36, Fabp4, Fabp5, galectin-3 (Lgals3) and lysosomal cathepsins (Ctsd), and are mainly in charge of clearing dead adipocytes and coordinating lipid handling (42, 44, 113). Both MMacs and ResVAMs are localized near blood vessels, and interact with endothelial cells to maintain vascular integrity. Of note, the transcriptional profile of MMacs (TREM2, Gpnmb, MARCO, TIMD4) share some similarities with LAMs. However, MMacs are enriched in genes that regulate diverse metabolic pathways, not just lipid processing (112).

### 6.3 EVs as mediators of adipose tissue-liver crosstalk

EVs are nanosized biovesicles that are secreted by cells, which carry various molecules from their parent cells, including miRNAs, mRNAs and proteins. EVs have emerged as a pivotal mediator facilitating crosstalk between the liver and adipose tissue. Therefore, EVs released from adipose tissue help regulate insulin sensitivity in MASLD (114). Macrophages in the adipose tissue of MASH mice develop a pro-inflammatory, lipid associated phenotype. They secrete small EVs which contain fibrogenic miRNAs, such as miR-155 and miR-34a (13). These miRNAs downregulate PPARγ in HSCs, therefore driving HSCs activation and liver fibrosis. Importantly, anti-inflammatory macrophage derived small EVs exert protective effects against fibrosis both *in vivo* and *in vitro (*13). High level of miR-29a is also found in exosomes secreted by obese ATMs, which is then transferred into hepatocytes and causes insulin resistance (115). miR-29a targets PPAR-δ, and PPARδ therapy partially rescues miR-29a-induced insulin resistance. Therefore, ATM-derived EVs directly link adipose dysfunction to hepatic malfunction in MASLD.

### 6.4 Adipokines linking adipose tissue and the liver

Accumulating evidence shows that adipose tissue might be the origins of low-grade inflammation, and secrets various pro- and anti-inflammatory cytokines named adipokines (116). Both adipose tissue macrophages and liver macrophages are key sources and targets of adipokines, regulating systemic metabolism and hepatic inflammation. Adiponectin, an adipokine, boosts insulin sensitivity and fights inflammation. In diet-induced obese mice, adiponectin prevented inflammation and insulin resistance, even though the mice still developed fatty liver. To be specific, hepatic adiponectin signaling blocks TNF-α-stimulated MCP-1, thus reducing macrophage infiltration and hepatic inflammation (117). Retinol-binding protein 4 (RBP4) synthesized by adipocytes also contribute to the development of metabolic diseases, including liver disease. Adipocyte-specific transgenic mice that express RBP4 display elevated hepatic triglycerides, obesity and glucose intolerance when fed with high-fat diet (118). These transgenic mice have increased expressions of TNF-α, leptin as well as crown-like structures in the adipose tissue. RBP4-induced inflammation stimulates increased lipolysis in adipocytes, causing elevated circulating FFAs, thus enhancing hepatic triglyceride level. Resistin has also been suggested to play a role in the pathogenesis of MASLD (119). Adipose tissue and macrophages both release resistin, which plays a role in glucose homeostasis and lipid metabolism. In animal models, resistin mainly targets the liver. However, conflicting results exist as regard to the contribution of resistin to the pathophysiology of MASLD. Several studies have demonstrated divergent conclusions with either lower, elevated or unchanged resistin levels in MASLD (120–122). In human MASLD, fibrosis may serve as a pro-inflammatory profibrogenic adipokine and is associated with increased serum level of resistin (123).

### 6.5 Metabolic intermediates as signaling molecules

Metabolic intermediates derived from dysfunctional adipose tissue activate adipose tissue and liver macrophages, shaping their inflammatory phenotype. FFAs and succinate are two key metabolic intermediates that link adipose tissue dysfunction to hepatic pathology of MASLD. FFAs released from insulin-resistant white adipose tissue (WAT) represent the canonical driver of hepatic steatosis (124). Under physiological conditions, WAT releases FFAs during fasting, and the liver uses these FFAs for gluconeogenesis. However, in obesity and insulin resistance, adipocyte lipolysis is dysregulated, causing uncontrolled FFAs release. The liver re-esterifies excessive FFAs into triglycerides, leading to steatosis. Moreover, FFAs and their metabolic intermediates such as ceramides and diacylglycerols directly cause hepatocyte injury via endoplasmic reticulum stress (ERS), mitochondrial dysfunction and oxidative stress. For example, palmitate binds directly to TLR4-MD2 complex on CD11b^+^F4/80^low^ hepatic infiltrating macrophages, rather than KCs, triggering dynamin-mediated endocytosis and NOX2-dependent ROS generation (125). In addition, HFD-fed mice lacking NOX2 are less prone to develop hepatic steatosis.

**Figure 4:**
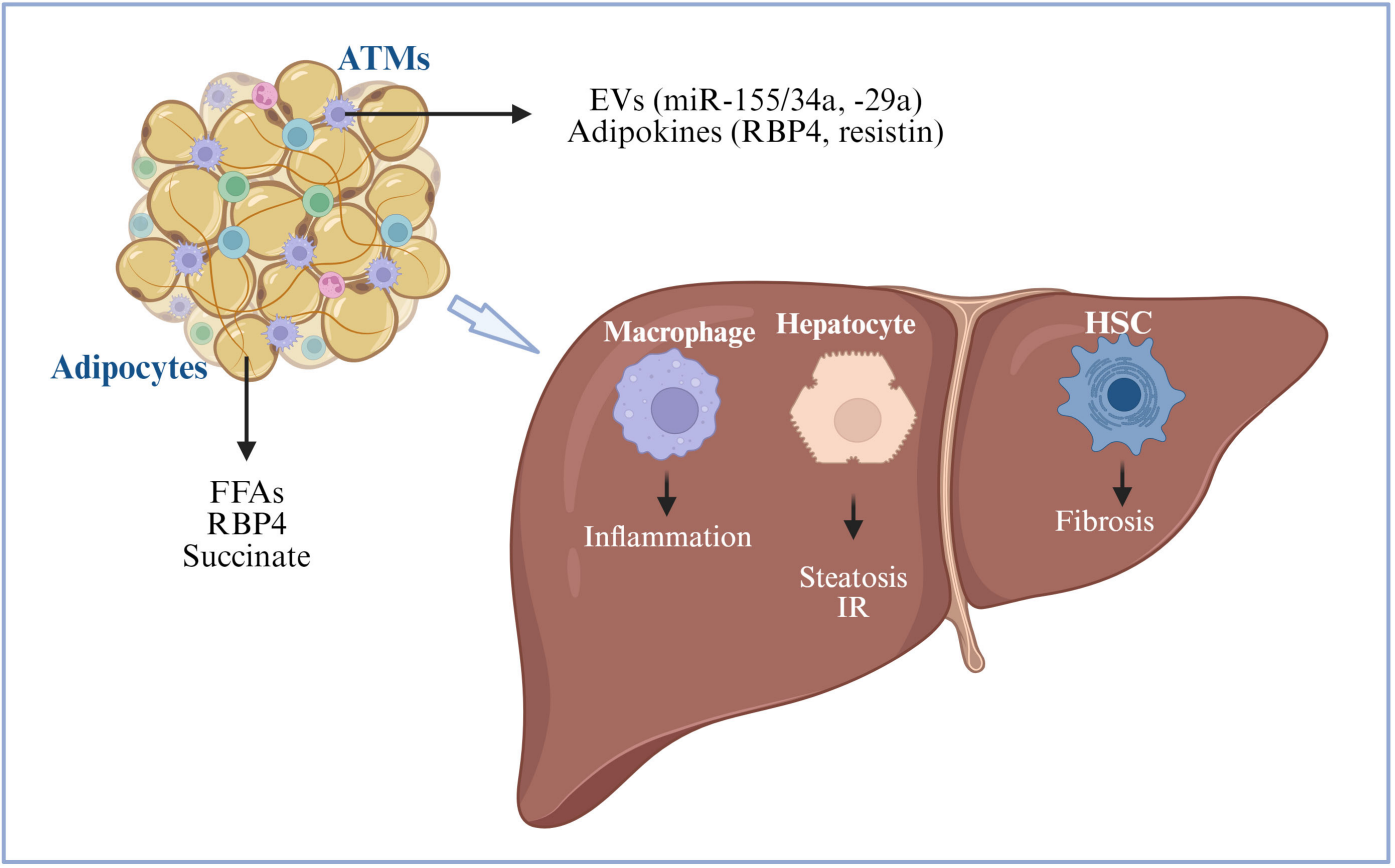
Adipose tissue-liver crosstalk in MASLD. Dysfunctional adipose tissue in obesity releases three classes of signals that promote MASLD progression. (1) Extracellular vesicles (EVs) derived from pro-inflammatory ATMs carry miR-155, miR-34a, and miR-29a, which induce hepatic stellate cell activation and hepatocyte insulin resistance. (2) Adipokines, notably RBP4, enhance hepatic steatosis and inflammation. (3) Metabolic intermediates, including free fatty acids (FFAs) and succinate, activate TLR4-NOX2-ROS signaling in hepatic macrophages and SUCNR1-dependent pro-fibrotic pathways in macrophages and HSCs. Created in BioRender. Sun, Y. (2026) https://BioRender.com/9x18m61.

Succinate, traditionally viewed as a tricarboxylic acid cycle intermediate, has gained recognition as an extracellular signaling molecule that modulates inflammation and fibrosis. Brown and beige adipose tissue oxidize circulating succinate via a UCP1 (uncoupling protein 1)-dependent mechanism, thereby lowering its levels. It was observed that succinate level was increased in the liver and brown/beige adipose tissue of UCP1 KO mice (85). Succinate then ligates to SUCNR1 in liver macrophages and stellate cells, promoting inflammation. To the contrary, increasing the content of brown/beige adipocytes antagonized SUCNR1-dependent inflammatory signaling, highlighting the extended role of brown/beige adipose tissue to defend MASLD and liver inflammation. The crosstalk between adipose tissue and the liver in MASLD is illustrated in **Figure 4**.

## 7 Skeletal muscle-liver crosstalk and MASLD

MASLD is intrinsically linked to widespread metabolic perturbations that extend to multiple organs. Among these, skeletal muscle has merged to play a vital role in the pathophysiology of MASLD, both as a target of metabolic dysfunction, and as a signaling organ that modulates hepatic outcomes. Skeletal muscle accounts for approximately 80% of system glucose disposal, and is central to whole-body metabolic homeostasis. In obesity and MASLD, however, the muscle microenvironment becomes a site of low-grade inflammation. Accumulation of pro-inflammatory macrophages disrupts local insulin signaling and systemic metabolic pathways, promoting hepatic steatosis and disease progression.

Foundational work by Patsouris established a causal association between skeletal muscle inflammation, macrophage activation as well as insulin resistance (126). Markers of skeletal muscle macrophages are increased in type 2 diabetes and correlate with insulin resistance. Moreover, muscle-specific overexpression of MCP1 is sufficient to recruit macrophages and impair muscle insulin sensitivity. Importantly, intra-muscular TNF-α expression is restricted to intramuscular leukocyte population, confirming that macrophages are the primary source of inflammatory cytokines. The above findings demonstrate that muscular microenvironment drives MCP-1 mediated recruitment of macrophages into skeletal muscle, which in turn exacerbates both local and system insulin resistance. Recent research has elucidated an endocrine pathway through which skeletal muscle interferon regulatory factor 4 (IRF4) and follistatin-like protein 1 (FSTL1) directly orchestrates liver pathology (127). FSTL1 is a critical myokine and is regulated by IRF4. Mice with muscle-specific deletion of IRF4 showed improved hepatic steatosis, inflammation and fibrosis when fed with MASH diet, while restoring FSTL1 expression could fully restore liver pathological changes. Co-culture studies also confirmed that FSTL1 exerted effect on hepatocytes, macrophages and HSCs through CD14 and DIP2A. In addition, human serum FSTL1 levels positively correlate with MASH progression. The above findings establish a direct association between skeletal muscle dysfunction and MASH pathophysiology. Given the central role of skeletal muscle dysfunction in MASH development, interventions that target muscle function hold particular promise. Among these, exercise, which profoundly improves muscle health and systemic metabolism, has been recognized as a cornerstone non-pharmacological strategy.

## 8 Exercise intervention of MASLD

The primary approach for MASLD patients without fibrosis involves lifestyle interventions, including changing diet, exercising and losing weight (49, 128). Studies show that in MASLD, weight loss of 3-5% could reduce hepatic steatosis, 5-7% lowers inflammation, and over 10% of weight loss even promotes fibrosis regression (129). A 52-week lifestyle program (hypocaloric diet plus exercise) confirmed that greater weight loss correlated with better histological outcomes, with the best results seen in patients losing ≥10% body weight (130). Nevertheless, a 48-week randomized trial found significant improvements in steatosis, inflammation, and ballooning, but not in fibrosis (131).

Besides lifestyle intervention-related weight loss, exercise alone is usually sufficient to alleviate MASLD. Current evidence supports structured exercise prescription for MASLD patients based on the FITT principle (Frequency, Intensity, Time, Type). The ACSM International Multidisciplinary Roundtable recommends at least 150 min/week of moderate-intensity or 75 min/week of vigorous-intensity physical activity (PA) for all patients with MASLD, with a preference for combined aerobic and resistance training (132). Exercise and Sport Science Australia (ESSA) also points out that as little as 135 min/week of aerobic exercise of at least moderate-intensity may be effective in reducing hepatic steatosis (133). A meta-analysis confirmed that combined aerobic and resistance training (3 sessions/week for 8–12 weeks) yielded most consistent benefits (134). Resistance training alone can be prescribed to patients who cannot tolerate aerobic exercise, with a minimum effective dose of 8–10 exercises at 60-80% of 1RM, at least 3 times per week for ≥12 weeks (135). Regarding exercise intensity, evidence regarding high-intensity interval training (HIIT) versus moderate-intensity continuous training (MIT) remains debated, when it comes to effects on intrahepatic lipid and ALT (136, 137). The ESSA position statement concluded that there appears to be no intensity-dependent benefit on hepatic steatosis, as long as recommended exercise volume is achieved (133). As regard to PA type, negative correlation is observed only between leisure-time PA and liver steatosis, but not between occupation-related PA or transportation-related PA and steatosis (138). In addition, increasing leisure-time PA is more effective in preventing fibrosis progression in men (138). While animal studies have demonstrated that excessive exercise promotes liver fibrosis (139), direct evidence from human-based studies remains largely lacking.

Subclinical chronic inflammation is a hallmark of MASLD, and exercise is now recognized as a potent lifestyle intervention that restores macrophage phenotypes, thereby reducing metaflammation and alleviating MASLD (15). During exercise, contracting skeletal muscles release anti-inflammatory cytokines, such as IL-1ra and IL-10, while suppressing TNF-α production and shifting the M1/M2 balance (15). Below summarizes current evidence from human and animal studies on exercise-induced macrophage modulation in MASLD.

### 8.1 Human studies

A systemic review has concluded that aerobic exercise induces hepatic fatty acid oxidation, activates AMPK, and influences key metabolic regulators such as PPAR-γ (140). However, evidence linking exercise to hepatic macrophage modulation is largely derived from studies examining ATMs and circulating inflammatory markers, because direct assessment of hepatic macrophages requires invasive liver biopsy.

In individuals with obesity, a condition closely associated with MASLD, WAT is dominated by pro-inflammatory M1-like macrophages, contributing to insulin resistance and metabolic complications. PA has been shown to re-establish the equilibrium between M1- and M2-like macrophages, thus mitigating metaflammation. In trained men subjected to 15 consecutive days of prolonged endurance exercise, a significant increase in CD163^+^ M2-like macrophages was found in gluteal and abdominal subcutaneous adipose tissue (141). However, a 12-week moderate-intensity exercise intervention without caloric restriction failed to alter serum levels of soluble CD163 (sCD163) in obese individuals (142). sCD163 is a novel marker of macrophage activation. It is increased in pathological conditions like obesity and type 2 diabetes, both of which are featured with activation of the monocyte-macrophage system (143). In contrast, dietary-induced weight loss significantly reduced sCD163 levels, partially normalizing them (142). The above two studies suggest that a higher exercise intensity or load may be required for maximal effects on adipose tissue macrophage phenotype. A large meta-analysis based on 75 datasets from exercise and immobilization research confirmed that exercise induces an immediate M1 surge followed by a sustained M2 activation with long-term training, a pattern consistent across species, sampling methods, and exercise types (144). This immunomodulatory effect supports the concept that exercise exerts direct anti-inflammatory actions by orchestrating a temporally regulated balance between pro-inflammatory and repair-oriented macrophage functions. In patients with MASLD, insulin resistance, myosteatosis and sarcopenia collectively accelerate progression of liver diseases. Exercise improves muscle mass, muscle quality, and metabolic level, thereby reducing systemic exposure to inflammatory mediators that influence hepatic macrophage activity (145).

### 8.2 Animal studies

Animal studies using rodent models of HFD-induced obesity and MASLD have provided insights in how exercise modulates macrophage phenotypes and functions. The effects of exercise intervention on macrophage polarization have been investigated in dietary-induced models of obesity and MASLD. In C57BL/6 mice fed with HFD for 6 weeks, treadmill exercise significantly protected against hepatic steatosis and normalized liver triglyceride accumulation (146). Of note, F4/80 gene expression in WAT was significantly elevated in the HFD mice whereas exercised HFD mice exhibited F4/80 levels comparable to the normal chow-fed controls. Similarly, in a mouse model of HFD- and fructose water-induced MASH, 16 weeks treadmill exercise reduced the quantity of KCs and downregulated hepatic CD36 and PPAR-γ (147). Ai et al. also demonstrated that downhill running promoted M2 macrophage polarization while inhibiting M1 macrophages, thereby attenuating chronic inflammation and hepatic steatosis in HFD mice (16). Exercise also alters intrahepatic immune cell composition by reducing BMDMs. In a MASH mouse model induced by western diet and carbon tetrachloride injection, 12 weeks of treadmill exercise decreased frequencies of hepatic F4/80^int^ CD11b^hi^ BMDMs (148). Since flow cytometry analysis revealed that the frequencies of BMDMs correlate positively with liver inflammation, steatosis and fibrosis, the above findings suggest that exercise suppresses MASH progression by altering immune cell composition in the liver. When exercise intensity is taken into account, HIIT is superior to MIT in reducing adiposity, enhancing glucose tolerance, and ameliorating hepatic steatosis, inflammation, and fibrosis. In a mouse model of MASH induced by high-fat high-carbohydrate diet, 16 weeks of HIIT resulted in a marked drop in hepatic MoMFs and bone marrow myeloid progenitor populations (149). Bulk liver RNA sequencing further confirmed that HIIT was more effective than MIT at reducing hepatic inflammation. The beneficial effects of exercise on MASLD are not limited to direct alterations in immune cell phenotypes in the liver and adipose tissue. They are also mediated by sophisticated inter-organ communication networks, particularly involving EVs.

### 8.3 EVs-mediated crosstalk and exercise effect

#### 8.3.1 Adipose tissue-liver

Adipose tissue dysfunction in obesity is linked to downregulation of AMPKα1, which promotes inter-organ communication by enhancing exosome biogenesis and secretion (150). AMPKα1 loss in adipocytes and WAT increases TSG101-driven exosome release. Under stimulation of palmitic acid, TSG101 sorts CD36 into exosomes, which are then taken up by hepatocytes, leading to steatosis and inflammation. Therefore, AMPK activators like metformin and exercise may be effective against MASLD. In addition, exercise confers protection against MASLD through exosomal signaling pathway. After 12-week aerobic exercise, exosomal miR-324 level was decreased and insulin sensitivity was improved. To be specific, adipose tissue-derived exosomal miR-324-5p enhances liver metabolic function via regulating ROCK1-mediated glucose and lipid metabolic signaling, as well as Akt-mediated survival signaling (151).

#### 8.3.2 Skeletal muscle-liver

Exercise training exerts profound hepatoprotective effect on MASLD, partly through skeletal muscle-derived EVs that deliver functional cargos to the liver. HIIT triggers the release of circulating exosomes enriched in miR-133a and miR-133b from the skeletal muscle (152). When sedentary mice receive exosomes collected from the trained mice, their insulin sensitivity is improved and plasma triglycerides are decreased. The above effects are mediated by direct targeting hepatic FoxO1, an insulin-regulated transcription factor controlling gluconeogenesis (153). Except classical exercise, remote limb ischemic conditioning also alleviates steatohepatitis in multiple mouse models of MASH via EVs-mediated effects (154). Limb ischemic conditioning causes muscle-liver transfer of small EVs loaded with miR-181d-5p, which suppresses hepatic nuclear receptor NR4A3 (155), a transcriptional regulator of metabolism and inflammation (154). Importantly, circulating EVs from human volunteers undergoing remote limb ischemic conditioning recapitulated anti-MASH benefits in animal models. Therefore, both exercise and remote limb ischemic conditioning could exert hepatoprotective effects by transient local ischemia and hypoxia, which serve as stimulus for EVs release. Conversely, the beneficial effects of exercise on metabolism are dose-dependent. Chronic overtraining could induce adverse muscle-liver crosstalk and lead to hepatic fibrosis (139). Excessive exercise in mice causes lactate build-up in the skeletal muscle, which drives SH3 domain-containing 3 (SORBS3) lactylation and package of F-box protein 2 (FBXO2) into a specialized class of small EVs termed “lactate bodies”. Lactate bodies could induce apoptosis of hepatocyte, as well as HSCs activation. The above study reveals that skeletal muscle could serve as both a source of protective signals and potential conduit of pathological signals. Therefore, a deeper understanding of how exercise modulates MASLD progression in a dose- and context-dependent manner represents a critical avenue for future investigation.

### 8.4 Myokine-mediated skeletal muscle-liver signaling and exercise effect

As an endocrine organ, skeletal muscle secrets a wide range of myokines, which are bioactive peptides mediating inter-organ communication (14). Accumulating studies have shown that exercise enhances the secretion of specific myokines that directly act on the liver, where they modulate glucose and lipid metabolism, reduce inflammation, and suppress fibrosis (156).

#### 8.4.1 Irisin

Irisin is an exercise-inducible myokine, which is proteolytically cleaved from fibronectin type III domain-containing protein 5 (FNDC5). Circulating irisin is significantly lower in MASLD patients than in healthy people (157). In a mouse model of HFD-induced MASLD, exercise training improved hepatic steatosis and fibrosis, and simultaneously increased irisin levels in both blood and muscle, suggesting that irisin may carry some of the protective effects of exercise (158). At the molecular level, irisin competitively binds to myeloid differentiation protein 2 (MD2), blocking the MD2-TLR4 pathway and thereby suppressing the downstream inflammatory response (158). Since TLR4 is highly expressed on KCs and its activation promotes M1 polarization in the liver, irisin-mediated MD2/TLR4 blockade likely contributes to suppression of pro-inflammatory macrophage activation during exercise. In addition, Exercise-induced irisin also regulates hepatic lipid metabolism via the PGC-1α/FNDC5 pathway (159).

#### 8.4.2 IL-15

The role of IL-15 in MASLD is context-dependent and vary with disease stages and animal models. In a 16-week HFD model to mimic early MASLD, IL-15 deficiency suppresses HFD-induced accumulation in the liver, and reduces expression of chemokines CCL2, CCL5 and CXCL10 (160). *In vitro*, IL-15 stimulation induces gene expressions of chemokines in wild-type, but not IL-15Rα lacking hepatocytes. Conversely, in a more severe MASH model, deficiency of IL-15 enhances fatty acids uptake into the liver, thus exacerbating fatty liver (148). These divergent findings may reflect differences in disease severity, IL-15 cellular sources, or the balance between anti-adipogenic effects of IL-15 and its immunomodulatory functions. In the latter study, exercise-induced IL-15 upregulation in the skeletal muscle was found to suppress MASH progression, an effect mediated by reduced intrahepatic bone marrow-derived macrophages (F4/80^int^CD11b^hi^ cells) (148).

#### 8.4.3 IL-6

IL-6 is typically considered as a pro-inflammatory cytokine, even though evidence also shows that production of IL-6 from the skeletal muscle usually exerts anti-inflammatory effects (161). For example, caffeine causes skeletal muscle to release IL-6, which leads to a 10-fold increase in IL-6 levels in the circulation. As a result, signal transducer and activator of transcription 3 (STAT3) is activated and MASLD is improved (162). The effect of IL-6 on macrophage accumulation is origin-dependent. IL-6 released by the adipose tissue enhances macrophage infiltration, while IL-6 derived from skeletal muscle or myeloid cells show the opposite effect (163). Following exercise, the circulating levels of IL-6 increase exponentially, and can reach up to 100-fold. This exercise-driven rise in plasma IL-6 is subsequently accompanied by elevated anti-inflammatory cytokines, including IL-1ra and IL-10 (164). Exercise training also exerts protective effect against MASLD via IL-6. STAT3 is abnormally activated in insulin resistance, and rats intervened with aerobic exercise training exhibited lower STAT3 activation, an effect mediated by IL-6 (165).

#### 8.4.4 Sclerostin

Sclerostin (SCL), primarily known as an osteocyte-derived inhibitor of bone formation, has also been proposed as a myokine involved in metabolic disorders (166). In hyperlipidemic conditions, SCL aggravates both skeletal muscle insulin resistance and hepatic steatosis. The mRNA level of SCL is increased in the myocytes of obese patients, as well as in the skeletal muscle of HFD-fed mice (166). At the molecular level, SCL enhances phosphorylation of mammalian target of rapamycin (mTOR) and suppresses autophagy markers, therefore causing ERS. Circulating SCL levels have been shown to decrease following exercise training, and even small changes in SCL concentration can influence metabolism (167). Therefore, it is plausible to speculate that exercise training might alleviate MASLD via modulating SCL.

**Figure 5:**
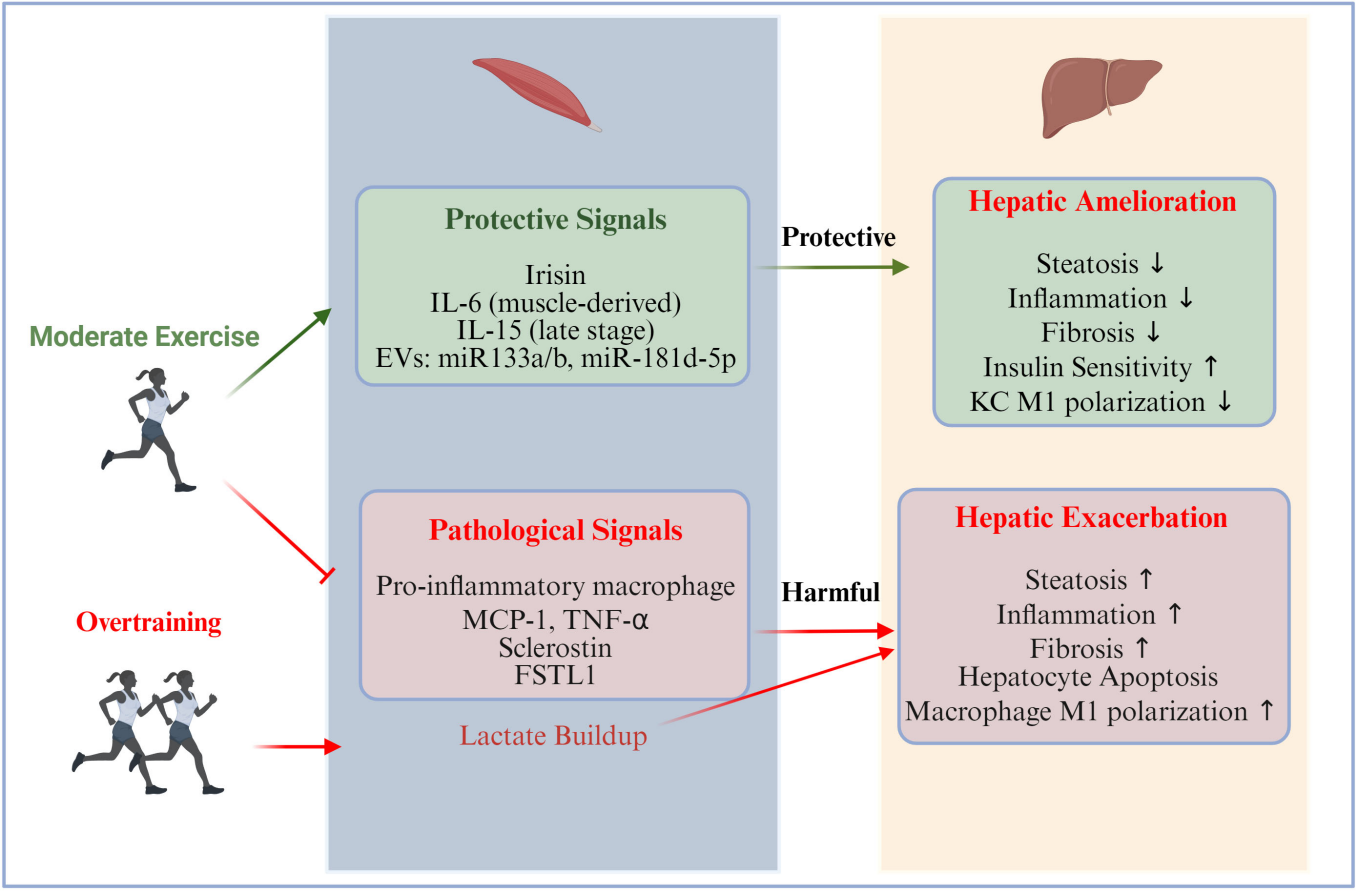
Exercise modulates MASLD/MASH via muscle-liver crosstalk. Exercise regulates MASLD/MASH progression through muscle-liver crosstalk. Moderate exercise promotes protective myokines/EVs (green arrows) that ameliorate hepatic pathology, while chronic overtraining or obesity triggers pathological signals (red arrows), including pro-inflammatory factors and FSTL1, that exacerbate liver injury. Created in BioRender. Sun, Y. (2026) https://BioRender.com/9x18m61.

The key findings from human and animal studies on exercise-regulated macrophage polarization, infiltration, and function are compiled in **Table 2**. **Figure 5** illustrates how exercise modulates MASLD/MASH via muscle-liver crosstalk.

**Table 2:** Exercise interventions targeting macrophage polarization and inflammation in MASLD: summary of direct evidence.

Reference	Species (model)	Detailed exercise intervention method	Exercise effects
Kawanishi, 2018 (147)	Mouse (HFF-induced MASH)	16 wk treadmill, 15–20 m/min, 60 min/d, 5 d/wk	↓ hepatic resident macrophages, ↓ CD36 & PPAR-γ in liver/macrophages
Tsutsui, 2023 (148)	Mouse (western diet and carbon tetrachloride-induced MASH)	12 wk treadmill, 60 min/d, 5 d/wk	↓ liver BMDMs
Fredrickson, 2021 (149)	Mouse (HFHC-induced MASH)	16 wk HIIT vs. MIT	HIIT > MIT: ↓ F4/80^int^CD11b^hi^ inflammatory macrophages in liver, ↓ myeloid progenitors in bone marrow
Gehrke, 2019 (173)	Mouse (HFHC-induced MASLD)	4 wk voluntary wheel running	↓ hepatic steatosis, ↓ macrophage infiltration
Baynard, 2012 (146)	Mouse (HFD-induced steatosis)	6 wk treadmill, 12 m/min, 12% grade, 40 min/d, 5 d/wk	↓ F4/80 expression in WAT, ↓ hepatic steatosis
Li, 2022 (174)	Mouse (HFD-induced steatosis)	16 wk swimming, 60 min/d, 5 d/wk	↑ liver macrophage migration inhibitory factor, ↓ lipotoxicity & JNK activation
Linden, 2014 (175)	Rat (OLETF)	12 wk treadmill: moderate vs. vigorous	moderate & vigorous: ↓ M1 polarization (CD11c, IL-1β); moderate: ↑ M2 polarization (CD206); no change in total macrophage markers (CD68, F4/80)
Oliveira, 2013 (176)	Rat (diet-induced obesity)	Acute exercise (single bout)	Phenotypic switch from M1- to M2-macrophages in WAT, improved insulin signaling
Zhang, 2025 (177)	Mouse (*db/db*)	8 wk treadmill, 60% Vmax, 60 min/d, 6 d/wk	↓ TWIK2-mtDNA-NLRP3 in hepatic macrophages, switch from M1- to M2-macrophages
Castaño, 2020 (152)	Mouse	5 wk HIIT treadmill, 18–26 m/min, 2min-2min cycle, 25°grade	↑ muscle exosomes (miR-133a/b-enriched), ↑ insulin sensitivity, ↓ hepatic FoxO1
Sahl, 2024 (141)	Human	15 consecutive days of prolonged endurance cycling (7–9 h/d)	↑ CD163^+^ M2 macrophages in gluteal & abdominal subcutaneous adipose tissue

Upward/downward arrows denote increase/decrease, respectively.

### 8.5 Exercise-metabolites crosstalk in macrophage repolarization

**Figure 6:**
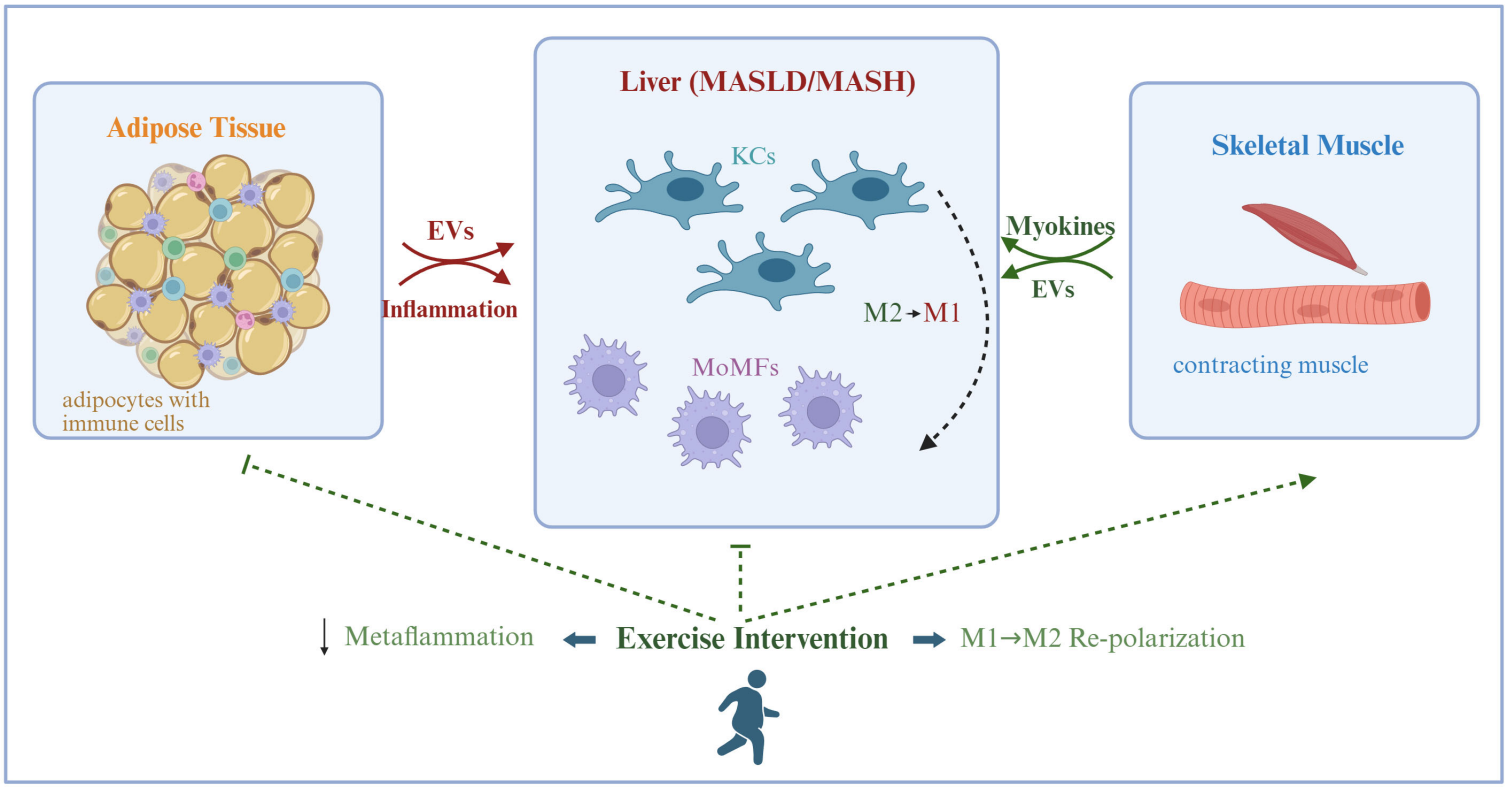
Schematic diagram of exercise-mediated multi-organ crosstalk in MASLD. Under pathological conditions, dysfunctional adipose tissue releases pro-inflammatory adipokines and extracellular vesicles (EVs), which promote hepatic inflammation and fibrosis. Concurrently, in the liver, resident Kupffer cells (KCs) undergo dysfunctional self-renewal and increased death, while infiltrating monocyte-derived macrophages (MoMFs) accumulate and drive steatohepatitis and fibrosis. Upon exercise intervention, contracting skeletal muscle secretes protective myokines and EVs that communicate with the liver to dampen inflammation, and adipose tissue reduces the release of pathogenic factors. The liver promotes M1-to-M2 macrophage re-polarization, reshaping intrahepatic immune landscape. Therefore, exercise acts as a systemic integrator to combat metaflammation in MASLD. Created in BioRender. Sun, Y. (2026) https://BioRender.com/9x18m61.

The recognition that metabolic intermediates such as lactate and succinate actively regulate macrophage polarization has opened new therapeutic avenues for MASLD. Preclinical evidence demonstrates that targeting lactate production via inhibition of hexokinase 2 (78, 80), or targeting lactate uptake via blocking monocarboxylate transporter 1 (79) attenuates hepatic inflammation and fibrosis. Similarly, inhibiting the succinate-SUCNR1 axis reduces fibrotic progression in MASH models (168). These findings suggest that pharmacological modulation of metabolite-driven pathways holds therapeutic promise. In addition, exercise intersects with these pathways in an integrated manner. A newly published study based on a murine MASLD model demonstrated that 8-week HIIT reversed HFD-induced lactate accumulation in circulation and the liver (169). The lactate level was also found positively correlated with hepatic fibrosis marker. At the molecular level, HIIT suppressed lactate production by downregulating lactate dehydrogenase A, while at the same time enhancing lactate clearance through MCT1. The above finding indicates that exercise alleviates pathological lactate accumulation through remodeling hepatic lactate metabolism, and might drive macrophage repolarization in MASLD. When it comes to succinate signaling, the story seems more complexed. Succinate released by skeletal muscle during acute exercise activates SUCNR1 in muscle macrophages, with M2 macrophages exhibiting high levels of SUCNR1 mRNA (170). This stands in contrast to the pro-inflammatory role of succinate-SUCNR1 signaling in hepatic macrophages as described in 5.3.3. Therefore, succinate might exert opposing effects on macrophages depending on tissue context and specific cell types. Nevertheless, it should be acknowledged that direct evidence regarding the effects of chronic exercise on succinate, SUCNR1 signaling, as well as subsequent impact on hepatic macrophage polarization remains limited. Further investigations are needed to explore whether exercise training consistently modulates succinate-SUCNR1 axis in the liver. We have included a schematic diagram that summarizes the coordinated actions of exercise on hepatic macrophages, adipose tissue, skeletal muscle and the liver in MASLD (**Figure 6**).

## 9 Conclusions and future perspectives

In this review, we have provided a systematic framework that integrates three previously separate lines of investigation: (1) hepatic macrophage heterogeneity and polarization, (2) adipose tissue-derived pro-inflammatory signals, and (3) skeletal muscle-derived anti-inflammatory myokines and EVs. To our knowledge, this is the first review that explicitly positions exercise as a systemic integrator, which rewires communication among multiple organs to reprogram hepatic macrophage function. Our review explains how exercise simultaneously dampens adipose-derived pro-inflammatory inputs while amplifying muscle-derived protective signals. In this review, we summarized the key role of hepatic macrophages in the pathogenesis of MASLD, with an emphasis on their inflammatory and non-inflammatory functions, their distinct heterogeneity, and involvement in inter-organ crosstalk with adipose tissue and skeletal muscle. It has become evident that macrophages are not merely amplifiers of liver inflammation, but are deeply integrated into metabolic regulation, fibrosis progression, and tissue repair.

We have now acquired sufficient information about macrophage subsets from single-cell studies, including LAMs, SAMs, KCs and MoMFs. However, very little is known regarding how exercise affects these specific subsets. The field has moved beyond the simple M1/M2 classification, but exercise research has not caught up. Mechanistic studies on macrophages are plentiful, but very few exercise studies use the same cutting-edge tools like scRNA-seq or fate mapping. This gap is large and needs to be addressed.

In order to guide future investigations, we would like to propose two testable hypotheses:

1. Exercise preferentially targets MoMFs rather than embryo-derived KCs, reducing their hepatic infiltration and inflammatory activation. This hypothesis can be tested using KC fate-mapping combined with scRNA-seq in a model of MASLD intervened with exercise training.
2. Different exercise modalities exert distinct effects on hepatic macrophage subsets, and the response of macrophages are disease stage-dependent. We predict that in early MASLD (steatosis with mild inflammation), HIIT is more effective than MIT in suppressing the expansion of LAMs. In contrast, during MASH with fibrosis, HIIT is superior to MIT in promoting LAMs expansion, which favors fibrosis resolution.

In a word, macrophage biology has advanced rapidly, while exercise research has lagged behind. This review highlights the gap and suggests future researchers to use modern tools to understand how exercise, a lifestyle intervention, reprograms liver macrophages in MASLD.
